# Inspiring academia in low- and middle-income countries

**DOI:** 10.1016/j.bas.2023.102689

**Published:** 2023-10-06

**Authors:** Adam Vacek, Chandrasekaran Kaliaperumal

**Affiliations:** The University of Edinburgh Medical School, Edinburgh, UK; Department of Clinical Neuroscience, Royal Infirmary of Edinburgh, UK

**Keywords:** Neurosurgery, Publication, LMIC, Barriers, Academia, Facilitators

Dear Editor in chief,

We read the article by Koester et al. (2023) with interest. The article well highlighted many of the barriers to neurosurgical publishing in low- and middle-income countries (LMCIs), such as costs of open access publishing, lack of equipment and experienced staff, and limited availability of access to existing scientific literature. We would like to highlight a few other barriers to academic development in LMICs and provide thoughts that would benefit the wider audience. Promoting academia in LMICs is crucial for the overall progress of the medical field worldwide, through the sharing of valuable research surrounding diagnosis and management of neurosurgical conditions in resource-restricted areas (Koester et al., 2023). We would like to highlight the following areas as opportunities to foster academic growth.

## 1 Resources and funding

One of the mentioned barriers to academic development is the need for more equipment and staff. Investments are needed to establish programs for aspiring researchers, such as workshops on research methodologies, data analysis and statistics, scientific writing, and publication ethics. This could be achieved through governmental or international federation support or partnerships with universities or research institutions. Furthermore, grants, scholarships, and research fellowships from these bodies would help combat the costs of neurosurgical publication and research, some of which we have covered previously (Vacek et al., 2022). Koester et al. also rightly mention the issues with the accessibility of existing neurosurgical publications to researchers in LMICs (Koester et al., 2023). Partnerships with libraries, universities, and other organisations allowing free or subsidised access to scientific literature, databases, and reference materials could help with academic material accessibility.

## 2 Aspiring authors

In academically active countries, aspiring researchers have numerous pathways to finding mentors who can assist them with developing their academic skills. Establishing mentorship programs connecting experienced researchers with emerging scholars could be done through collaborative research projects between researchers from LMICs and those from high-income countries (Citrin et al., 2017). These programs could also provide researchers from LMICs with opportunities to start publishing in international journals. Similar outcomes could be achieved through dedicated international partnerships between academic institutions in LMICs and those in high-income countries. Additionally, fostering the development of local neurosurgical journals, and supporting them in meeting international publishing standards and getting indexed in reputable databases could provide local researchers with additional opportunities to publish their work and develop the skills required for conducting rigorous neurosurgical research. This could also help mitigate the centralisation issues of neurosurgical publishing (Vacek et al., 2022; Qazi et al., 2023). Furthermore, empowering medical students can mould the future of academics and break the barriers beyond boundaries (Minta et al., 2023).

## 3 Wider publication barriers

Language can be an additional barrier to academic publishing for many authors in LMICs. Providing accessible translation services for creating new neurosurgical work and reviewing existing international literature could be essential to fostering academic growth in these countries (Jain et al., 2022). In addition, promoting open access publishing can, combined with tackling the funding issues of open access highlighted above, increase the accessibility of international neurosurgical research to LMICs, and allow the wider neurosurgical community to learn from the findings of researchers from LMICs. Furthermore, supporting the development of local peer review networks could contribute to the overall quality of research, help develop local connections among researchers, serve as a first step in launching a local neurosurgical journal, and increase advocacy for research ethics and integrity, ensuring studies conducted in LMICs adhere to international ethical standards, improving their chances for international recognition.

## 4 Awareness and advocacy

Raising awareness about the importance of academic development in LMICs and the benefits they can bring to the wider neurosurgical community is a crucial aspect of this topic. Advocating for efforts to establish funding and support for the above-described interventions could significantly impact international neurosurgical knowledge and improve patient care worldwide (Koester et al., 2023; Richards and James, 2004). Local and international governments can support awareness and advocacy and create initiatives through policies, grants, and research funding. For instance, incentives and recognition systems could be established to acknowledge and reward researchers in LMICs for their contributions to neurosurgical knowledgebase through awards, honours, and career advancement opportunities. Similarly, international federations like the World Federation of Neurosurgical Societies (WFNS), the European Association of Neurosurgical Societies (EANS), and the Congress of Neurological Surgeons (CNS) have an important role to play in encouraging neurosurgical academic publications in LMICs. However, it should be noted that governments and international federations should seek to engage with all of the issues mentioned in this letter, not only awareness and advocacy.

By implementing these suggestions, the neurosurgical community can work together to create an environment conducive to academic development in LMICs, subsequently improving healthcare and advancing neurosurgical scientific knowledge worldwide. Everybody has a role, from individuals to research teams, academic institutions, international federations, and governments.

Sincerely,

Adam Vacek and Chandrasekaran Kaliaperumal.

## Declaration of competing interest

The authors declare that they have no known competing financial interests or personal relationships that could have appeared to influence the work reported in this paper.
